# Environmental Lead Exposure and Influenza and Respiratory Syncytial Virus Diagnoses in Young Children: A Test-Negative Case-Control Study

**DOI:** 10.3390/ijerph17207625

**Published:** 2020-10-19

**Authors:** Marina Oktapodas Feiler, Mary T. Caserta, Edwin van Wijngaarden, Kelly Thevenet-Morrison, Dwight J. Hardy, Yan Victoria Zhang, Ann M. Dozier, B. Paige Lawrence, Todd A. Jusko

**Affiliations:** 1 Department of Environmental Medicine, School of Medicine and Dentistry, University of Rochester, 601 Elmwood Ave, Rochester, NY 14642, USA; edwin_van_wijngaarden@urmc.rochester.edu (E.v.W.); paige_lawrence@urmc.rochester.edu (B.P.L.); todd_jusko@urmc.rochester.edu (T.A.J.); 2 Department of Pediatrics, School of Medicine and Dentistry, University of Rochester, 601 Elmwood Ave, Rochester, NY 14642, USA; mary_caserta@urmc.rochester.edu; 3 Department of Public Health Sciences, School of Medicine and Dentistry, University of Rochester, 265 Crittenden Blvd, Rochester, NY 14620, USA; kelly_thevenet-morrison@urmc.rochester.edu (K.T.-M.); ann_dozier@urmc.rochester.edu (A.M.D.); 4 Department of Microbiology and Immunology, School of Medicine and Dentistry, University of Rochester, 601 Elmwood Ave, Rochester, NY 14642, USA; dwight_hardy@urmc.rochester.edu; 5 Department of Pathology and Laboratory Medicine, School of Medicine and Dentistry, University of Rochester, 601 Elmwood Ave, Rochester, NY 14642, USA; victoria_zhang@urmc.rochester.edu

**Keywords:** flu, RSV, Pb, blood lead, pediatric, respiratory infection, sex-specific influenza, immunotoxicology, infectious disease, exposure, environment

## Abstract

Experimental and epidemiological evidence suggests that environmental toxicants may influence susceptibility to influenza and respiratory syncytial virus (RSV). The objective of the present study was to estimate the association between blood lead concentrations and the odds of child influenza or RSV infection. A test-negative, case-control study was conducted among 617 children, <4 years of age, tested for influenza/RSV from 2012–2017 in Rochester, NY. There were 49 influenza cases (568 controls) and 123 RSV cases (494 controls). Blood lead concentrations reported in children’s medical records were linked with influenza/RSV lab test results. Covariables were collected from medical records, birth certificates, and U.S. census data. In this sample, evidence of an association between blood lead levels and RSV or influenza diagnosis was not observed. Children with a lead level ≥1 μg/dL vs. <1 μg/dL had an adjusted odds ratio (aOR) and 95% confidence limit of 0.95 (0.60, 1.49) for RSV and 1.34 (0.65, 2.75) for influenza. In sex-specific analyses, boys with lead concentrations ≥1 μg/dL vs. <1 μg/dL had an aOR = 1.89 (1.25, 2.86) for influenza diagnosis, while the estimates were inconsistent for girls. These results are suggestive of sex-specific associations between blood lead levels and the risk of influenza, although the sample size was small.

## 1. Introduction

The burden of influenza viruses and respiratory syncytial virus (RSV) in the United States is substantial: nearly 20,000 children under the age of 5 years are hospitalized annually due to influenza-related complications and 40% of all pediatric deaths due to influenza were in children under 5 years of age [1]. Additionally, RSV accounts for over two million annual outpatient visits among children. A recent systematic review and meta-analysis observed the annual hospitalization rate of 4.4 per 1000 for RSV among children <5 years of age [2], equating to over 57,000 annual hospitalizations in the United States alone.

The immune system plays a critical role in preventing infectious disease at all ages, including childhood. Greater infectious morbidity, as well as potentially insufficient responses to childhood vaccinations have been linked to certain demographic, socioeconomic, and clinical factors, as have environmental factors such as exposure to polychlorinated biphenyls [3,4,5,6,7] and perfluorinated compounds [8,9]. Studies of heavy metals, such as arsenic, lead and cadmium and their effects on the perinatal immune system have [10,11,12,13,14,15] shown inverse relationships between exposure and the number of CD4+ and CD8+ T cells, B cells, and non-specific IgG concentrations [10,11], however, results are inconsistent. Exposure to lead is particularly a global public health concern given its ubiquity of exposure, specifically for children since they experience relatively higher blood lead levels than adults and are more susceptible to its effects. The current guidelines by the Centers for Disease Control and Prevention (CDC) have set an action level for blood lead levels at 5 µg/dL [16]. Children with these levels need to be monitored with potential for remediation of the home. A blood lead level of 10 µg/dL is now considered lead poisoning for children. Potential new guidelines by the CDC will lower this action level to 3.5 µg/dL. Given the studies that have examined this relationship, the immunological health effects of low-level lead exposure in infants and young children have not been well-established [11,12,13,14,15]. The response to the influenza virus and the influenza vaccine is T-cell dependent; it is hypothesized that this response may be adversely impacted by exposure to lead caused by lead-induced toxicity to the immune system [10,17]. No previous studies have investigated the effects of lead exposure on respiratory disease as a potential consequence of a diminished immune response.

The objective of the proposed study is to assess whether a higher concentration of blood lead during early life is associated with an increased risk of influenza and/or RSV diagnosis in children less than 4 years of age.

## 2. Materials and Methods

### 2.1. Case and Control Selection

Study subjects were children tested for influenza and RSV via molecular diagnostic methods at the University of Rochester Medical Center (URMC), in Rochester, New York, between 2012 and 2017. URMC utilizes the real-time polymerase-chain-reaction (PCR) method for detecting these two viral pathogens. The testing laboratory at URMC is permitted by the New York State Department of Health, Clinical Laboratory Improvement Amendments (CLIA)-permitted, and accredited by the College of American Pathologists to perform procedures including PCR for influenza/RSV. In addition to in-house quality assurance programs, the laboratory also follows standard operating procedures that require review and approval by a laboratory director, documentation of on-going proficiency, and all personnel to demonstrate competency on an on-going basis. Nasopharyngeal swabs were performed by medical care providers based on clinical judgment for suspected respiratory infection. Children included in the study were those <4 years of age at the time of testing. This age range was selected owing to the high prevalence of RSV in younger populations (<1 year of age), and because the risk of influenza-related complications is greatest among children under the age of 5 years (specifically under the age of 2 years). This study examined children who presented to URMC during the respiratory season (October 1–May 31), and were tested for influenza and/or RSV either due to symptomatology or because they presented to the emergency department during a high exposure season to viral respiratory pathogens. Study protocols were reviewed and approved by the research subjects review board (RSRB) at URMCRSRB (RSRB00070915). Cases were defined as children with positive influenza or RSV tests, and controls were defined as children with negative influenza or RSV tests. Two different testing platforms were included in this study. The first tests for influenza A and B, and RSV infection. The second is a full respiratory viral panel, including influenza A and B, RSV, and seven other respiratory viruses. Children could be negative for both influenza and RSV, positive for both, or positive for any other combination of respiratory pathogens. The tests used at URMC are considered the gold standard for viral respiratory diseases, with a high specificity of 95.9%, and relatively high sensitivity of 77.2%, while other studies have determined sensitivity levels ranging from 67–91% depending on the influenza type and age range of the individuals tested [18].

### 2.2. Blood Lead Determination

New York State requires lead screening by primary healthcare providers for children aged 6 months to 6 years of age [19]. Providers are expected to screen for lead at each well-child visit or at least annually if the child does not have a well-child visit [19]. In order to quantify exposure, children’s medical records were abstracted to identify extant blood lead measurements. Existing blood lead measurements entered into children’s medical records came from laboratory tests ordered during previous visits to URMC, and concentrations were determined at the URMC laboratory. Whole blood lead concentrations were determined using graphite furnace atomic absorption spectrometry. For venous lead measurements, 3 mL of whole blood was collected in a lead-free tan tube, and for capillary lead measurements, patients’ hands were washed and dried, and 0.5 mL blood was collected by finger prick in an EDTA containing microtainer. The atomic absorption spectrometry method has a reportable range of 1–60 µg/dL and a limit of quantification (LOQ) of 1 µg/dL. Venous blood lead samples measured at greater than 60 µg/dL are reanalyzed, and capillary samples with results greater than five µg/dL arere-drawn using venipuncture when possible to rule out contamination. If the blood lead concentration is below the LOQ, it is reported by the lab as <1.0 µg/dL. In this study, both capillary and venous blood lead values were included in order to increase the sample size. The majority of children had multiple lead measurements. When multiple measurements were present, peak lead levels were utilized, estimated by using the maximum blood lead concentration among the multiple lead measurements.

### 2.3. Collection of All Other Covariables

**Figure 1:**
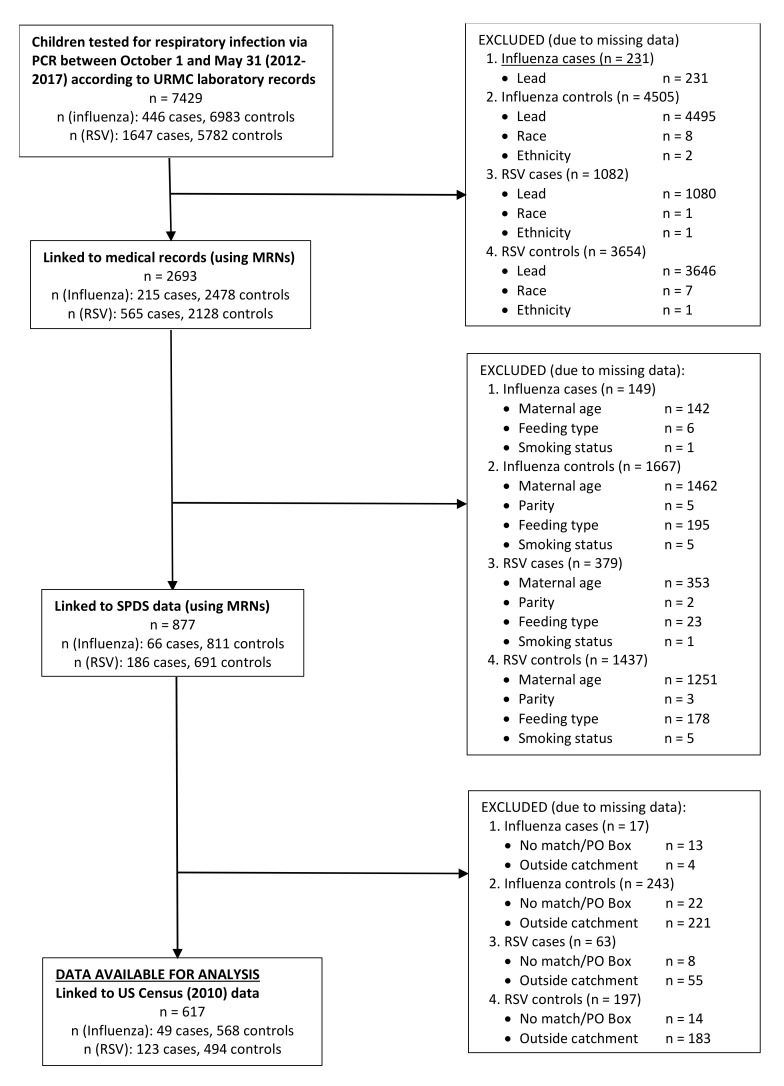
Flowchart Describing Collection of Influenza and Respiratory Syncytial Virus (RSV), Data Sources, Covariables of Interest, and Missingness for the Sample with Complete Data.

The majority of the *a priori* confounders considered in this study, child age at influenza/RSV test (months), child sex, child race (white, black, other), child ethnicity (Hispanic/non-Hispanic), child insurance status (private/public or self-pay), child blood lead measurements, addresses and zip-codes, influenza vaccination status, and respiratory season of testing (2012, 2013, 2014, 2015, or 2017) were obtained by linking children’s medical records with RSV/influenza test data using the name, date of birth, and medical record number (MRNs). Data linking was performed by utilizing the URMC system of Informatics for Integrating Biology and Bedside (i2b2). This system can pool together data on individuals who have medical records as part of the URMC EPIC system using medical record numbers (MRNs) (Figure 1). All maternal characteristics were obtained from childbirth certificates and questionnaires administered as part of the Statewide Perinatal Data System (SPDS). Data were linked using children’s MRNs, and data were only available for women who were delivered at Strong Memorial Hospital (SMH) (Figure 1). Maternal characteristics obtained by SPDS include maternal age (<20, 20–24, 25–30, 30–34, ≥35 years), maternal smoking (women were considered smokers if they reported smoking during pregnancy or 3 months prior to pregnancy), feeding type during hospitalization after delivery (formula only, breastfeeding only, both, or neither such as a feeding tube), and parity. Socioeconomic status (SES) was estimated by using the geographic information system (GIS) and spatial modeling techniques, which mapped and identified each child’s census tract using the ArcGIS program. Children’s addresses were mapped and geocoded to assess their census tract by using census mapping. After every child’s census tract was matched, average SES factors were determined by using data from the U.S. census bureau. SES factors considered area-level unemployment, area-level less than a college education (no four-year college degree obtained), area-level poverty status, and housing built before 1980 [20,21,22,23,24,25,26,27]. These variables were categorized by finding the median proportion of these variables in the census tracts included in the study (N = 190), individuals’ SES characteristics were then coded whether they resided in a tract that had above or below the median proportion in all tracts (Figure 1). The potential confounders discussed above were identified based on the extant literature concerning human immunotoxicity, and the final, minimally sufficient set of potential confounders were identified using graphical methods (i.e., directed acyclic graph (DAG)).

### 2.4. Statistical Analyses

#### 2.4.1. Descriptive Analyses

Univariate analyses were performed to describe the study sample and to compare cases and controls of influenza and RSV. Lead measures were only used if collection was prior to viral testing or within one month after testing to ensure temporality of exposure and outcome [28]. Any lead values collected beyond 1 month of viral testing were excluded, before selecting a peak lead value [28]. Differences between all covariables comparing children with and without available lead measures were also tested. All data management and statistical analyses were performed using the SAS software system (SAS Institute Inc., Cary, NY, USA; version 9.4). For all statistical tests, a *p*-value < 0.05 was considered statistically significant.

#### 2.4.2. Analyses of Lead and RSV/Influenza

Logistic regression models were fit to estimate the crude and adjusted odds ratio (aOR) and 95% confidence intervals (CI) of RSV or influenza diagnosis, using a set of pre-specified covariables. Separate crude and adjusted models were fit for each outcome, varying the parameterization of blood lead. Blood lead was parameterized as binary (above or below the LOQ, 1 µg/dL; above or below 3 µg/dL), and as a three-level variable; <LOQ, LOQ-3 µg/dL, and ≥3 µg/dL. Three µg/dL was used as a category to inform the potentially new action level for lead by the CDC is 3.5 µg/dL, as 3 µg/dL was the closest level that could be used with the whole integer data available for the present analysis. Logistic regression models were also fit to estimate the odds and 95% confidence intervals of peak lead with influenza diagnosis while considering an interaction term between blood lead and child sex.

#### 2.4.3. Secondary Analyses

Logistic regression models were fit to estimate the crude and adjusted odds ratios and 95% confidence intervals of RSV and influenza diagnosis stratified by child sex. Analyses were also conducted among the larger sample size (N = 2663), which included those participants with complete data on medical record covariables, (child age, race, ethnicity, insurance status, and respiratory season), but individuals with missing data on potential confounders from SPDS (maternal characteristics) and area-level SES characteristics were not excluded in these analyses.

#### 2.4.4. Sensitivity Analyses

Two sets of models were fit to test the robustness of the primary models for both RSV and influenza outcomes. The models were identical to primary models with some differences, which are highlighted in the numbered models to follow. Model set (1) analyzed venous blood lead measurements exclusively with influenza and RSV, given their greater reliability and accuracy compared with capillary measures, in order to examine whether combining these with capillary measures was appropriate. In model set (2) peak lead values were replaced by average lead values and models with influenza and RSV were re-run. When comparing peak and average lead values, the two were highly correlated, with a Spearman correlation r = 0.99, *p* < 0.001. Peak lead values were ultimately used in order to provide the most policy-relevant exposure. Additional models were run examining the association between lead and influenza while considering a smaller set of potential confounders. Adjusted logistic regression analyses were performed to determine the OR (95% CI) for BLLs and influenza while only considering covariables available in medical records; these include child sex, race, ethnicity, insurance status, and respiratory season.

### 2.5. Vaccination Information

A review of medical records was performed to collect influenza vaccination information. Medical records only listed the vaccination status of some children and had a missing status for others. Children all had a vaccination status tab in their medical records, some children had an influenza vaccination recorded, while all others had a missing value recorded. Thus, it was unknown whether children with a missing vaccination status had or had not received a vaccine. For this reason, estimating vaccine effectiveness was not possible.

## 3. Results

### 3.1. Sample Characteristics

The study sample consisted of 617 children <4 years of age with influenza/PCR tests from 2012–2017 with complete data from all data sources (Table 1). Among these children, 49 tested positive for influenza, and 568 tested negative for influenza while 123 had RSV, and 494 children were RSV negative. No child tested positive for both influenza and RSV. Among positive influenza cases, 38 (78%) were diagnosed with influenza A, and all influenza A and B cases were combined in order to enhance the sample size. The median age of children with influenza was 10.8 months and the median age for children with RSV was 6.0 months. When considering differences among children with peak lead values dichotomized at 3 μg/dL, children with higher lead values were more likely to have parent/guardian reported that identified as Black, Hispanic ethnicity, public health insurance, be female, have younger mothers who were nulliparous, have mothers who smoked during pregnancy or 3 months prior to pregnancy, and reside in a census tract with a high proportion of unemployment, less than college education, and housing built before 1980 (Table 1). In addition, when considering all children with an available viral test and available lead (n = 2663), compared with children with complete information (n = 617), children from the larger sample were older; all other characteristics were similar (Supplementary Table S1). These two samples were also compared with samples identical to each, except for the availability of blood lead. Overall, children with available lead values were older, were more likely to have parent/guardian reported white race and non-Hispanic ethnicity, while insurance status and sex differences were similar (Supplementary Table S1). In the reduced sample with complete covariable information (N = 1278), 617 had available lead values and 661 had no blood lead values available (Table 2). These two subgroups were compared in order to determine potential selection bias by available lead values. Children with available lead values appear to be older, identify as Black, have younger mothers, and reside in a census tract with a high proportion of unemployment, and less than a college education (Table 2).

### 3.2. Blood Lead Distribution

**Figure 2:**
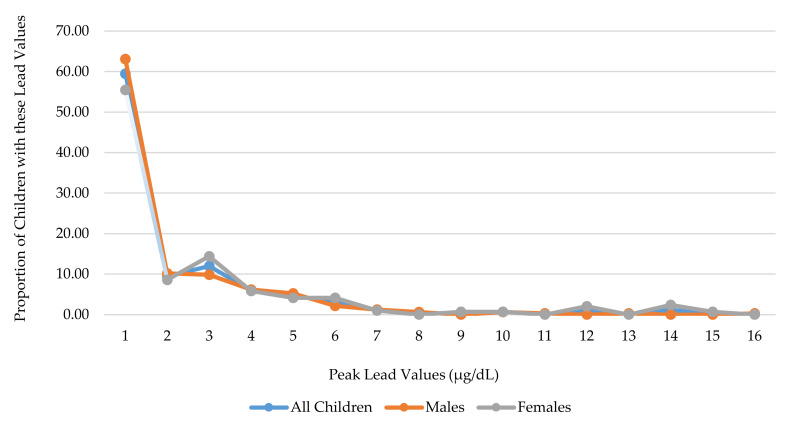
Distribution of Peak Lead Values (N = 617).

The available lead data, in both the reduced (N = 617) and larger samples (N = 2663) had log-normal distributions and were right-skewed, with the majority of children having a lead value at or below the limit of quantification (1 µg/dL) (Figure 2). The median lead value was <LOQ with an interquartile range from <LOQ to 2 µg/dL. Additionally, 5% of children had a concentration ≥5 µg/dL, and 2% had a concentration ≥ 10 µg/dL (Figure 2). Fifty-six percent of children had more than one lead measurement, 46% of children had one lead measurement, 37% had two lead measurements, and 13% had 3 lead measurements, ranging up to 11 lead measurements (N = 1), among the reduced sample.

### 3.3. Lead and RSV

Crude and adjusted analyses showed little evidence of an association between blood lead levels and RSV diagnosis, regardless of lead parameterization. The crude OR (95% CI) comparing children with a lead value above or below the limit of quantification (1 μg/dL) was 0.97 (0.65, 1.45). When considering lead and RSV among the larger sample, only controlling for medical record variables, there was a complete sample of 558 RSV cases, and 2105 controls (Table 3). Results from this analysis indicate a 19% greater odds of RSV diagnosis (95% CI: 0.98, 1.46) among children with higher blood lead levels. When adjusting for all covariables, children with a lead value ≥LOQ had 0.95 times the risk of RSV, compared with children who had a lead value <LOQ (aOR: 95% CI; 0.95: 0.60, 1.49) (Table 3).

### 3.4. Lead and Influenza

Crude and adjusted analyses suggested evidence of increased risk of influenza among children with higher, compared with lower lead levels (Table 4). Children with a lead level ≥LOQ had a 59% increased risk of influenza diagnosis (95% CI: 0.89, 2.85), compared with children who had a lead level <LOQ (Table 4). This association remained after full adjustment, however, the association was attenuated (aOR: 95% CI; 1.34: 0.65, 2.75) (Table 4). In addition, in models categorizing lead exposure, an increased risk of influenza diagnosis among higher levels of lead exposure compared with a level ≤1 µg/dL, those with a level of 1–3 µg/dL had 1.36 times the odds of influenza diagnosis, aOR (95% CI): 1.36 (0.52, 3.59) (Table 4). When considering lead and influenza among a larger sample and only controlling for medical record variables, there was a complete sample of 209 influenza cases and 2454 controls (Table 4). Children with a blood lead level ≥LOQ, compared with those with a level <LOQ had a 53% greater odds of influenza diagnosis (95% CI: 1.13, 2.08) (Table 4).

### 3.5. Secondary Analyses

Results of stratified analyses represented an increased risk of influenza diagnosis among males in the larger, minimally adjusted sample (LOQ split aOR: 95% CI; 1.89: 1.25, 2.86) (Table 5). Results for RSV were imprecise and close to the null, while results for influenza were consistently suggestive of an increased odds for males and females. However, results were more precise and represented a larger magnitude for males, as compared with females.

### 3.6. Sensitivity Analyses

Sensitivity analyses evaluating venous versus capillary blood lead measurements suggested venous blood measurements attenuate the effect of lead on influenza diagnosis. When comparing the LOQ split, the crude OR (95% CI) for venous blood lead measurements only (N = 38 cases) was 1.11 (0.57, 2.18) (results not shown). By including both blood types in the main analyses, the sample size increased (N = 49 cases) and an odds ratio of 1.59, 95% CI: 0.89, 2.85 was observed. Additional models fit to determine the OR (95% CI) for lead and influenza, using a reduced set of potential confounders, resulted in larger odds ratios and more precise estimates compared with the fully adjusted logistic regression models. Those with a BLL ≥LOQ had 1.93 times the odds of an influenza diagnosis, compared with those with a BLL < LOQ (95% CI: 1.02, 3.67) (Table 6). All other estimates were consistently larger compared with the fully adjusted models, suggestive of an increased risk of influenza with higher BLLs.

## 4. Discussion

The objective of the present study was to determine whether children with higher blood lead levels, due to environmental lead exposure, have an increased risk of influenza and RSV diagnosis, compared with children who have lower blood lead levels. Based on previous epidemiologic research suggesting reduced immune function with higher levels of lead exposure it was hypothesized that higher lead levels would be associated with an increased risk of infection [11,12,14,15,29].

This study did not observe an association between blood lead concentrations in young children and their infection risk to either RSV or influenza virus when considering both sexes together. RSV is very common in young children, as young as six months of age. Most lead measurements in the current study were obtained from children 1 or 2 years of age. Consequently, some RSV positive children with higher lead concentrations at a younger age may not have been considered, due to missing lead data. Additionally, given the volume of missing data, the sample size was greatly reduced when considering all covariables of interest. This reduced the study’s power to detect larger effect estimates, and likely produced the imprecise estimates observed. Post-hoc power calculations showed <20% power to observe the adjusted odds ratios reported in the reduced sample with complete data, when considering the LOQ split. For the larger, minimally adjusted sample, post-hoc power calculations showed 43% power to detect the LOQ split OR for RSV, and 81% power to detect the OR for influenza. To detect an OR = 1.59 for influenza, a sample size of 1920 participants (with 160 cases) would be needed to achieve at least 80% power.

Previous studies have reported associations between higher lead exposure and lower immune cell volumes [11,12,14,15,29]. However, whether reductions in these immune cell populations are enough to increase children’s risk for the disease remains to be determined. Six previous studies investigated lead exposure and immune cells in young children [11,12,13,14,15,29]. Of these six studies, four of these found reductions in immune cell populations among children with higher lead concentrations, or found negative correlations between lead and various immune cell populations [11,12,14,15]. One study examined blood lead levels and a variety of immune parameters, including antibody titers to the rubella vaccine [12]. The population of interest included 279 children 9–48 months of age, and age was the only covariate included in the analysis. The authors reported negative Spearman correlations with rubella antibody titers (r = −0.09), activated CD3 T cells (r = −0.04), and B cells (r = −0.05) [12]. An additional study examining 70 children 3–6 years old, assessed the associations between blood lead levels of ≥10 µg/dL vs. <10 µg/dL with T lymphocyte concentrations. Negative correlations were seen for children with high, compared with low lead levels for CD3 T cells (r = −0.203), CD4 T cells (r = −0.462), and CD8 T cells (r = −0.196) [11]. Although correlations in these studies were small to moderate, it is not yet well understood how change in different cell subpopulations can influence the immune system.

Studies using animal models have suggested that even small reductions in immune cell populations can have large impacts on the functioning of the immune system [30]. It is not yet established how large of a change in immune cells is necessary to negatively impact the immune system. Small declines in immune cell populations may or may not be enough to reduce the ability of the immune system to effectively protect the body against infection. It is possible that lead exposure does not measurably affect the percentage or number of immune cells but instead changes their function in a manner that skews or blunts their response to an immune challenge, such as infection or vaccination. Thus, investigating reductions in the frequency of cell populations is not adequate to understand the impact lead exposure has on the immune system.

The current study observed null associations between lead and RSV or influenza among the entire study sample. When stratifying results by sex, a statistically significant increased risk of influenza diagnosis among males with higher, compared with lower lead levels was observed. These results are only suggestive of an association between lead and influenza among males, as these analyses were exploratory given the small sample size after sex-stratification. Stratified results for RSV surrounded the null for both males and females, results were very inconsistent. This may be due to similar limitations surrounding RSV as with primary results. RSV results may be imprecise, given RSV is most common in the first year of life and very few children in the present study population had available blood lead levels before the age of one. Previous literature regarding lead toxicity is inconsistent with regard to sex differences in children, several different outcomes have been examined (i.e., immune function, cognition, and other neurodevelopmental effects), and the results have been inconsistent [14,31,32,33]. However, some experimental studies reported greater lead toxicity related to the immune system among males, compared with females. One study of mice found that young males have higher whole-body retention of lead than female mice [34]. Authors also observed an increased toxicity of lead acetate in young males, compared with young female mice [34]. An additional study observed reductions in thymic weights for both male and female mice, associated with exposure to lead acetate in drinking water during early life [35]. The authors reported greater thymic weight reductions in males, compared with females, although this difference was not statistically significant [35]. Thymic weight is important to consider as the thymus is an important organ for T cell development [36]. In addition precursor cells travel from bone marrow to the thymus where they mature into T cells, and bone is one of the major sites where lead is stored in the body [37,38]. Among the pediatric infectious disease literature, it has been observed that sex has a major impact on infectious disease [39]. Morbidity and mortality are greater among males, from infancy and into adulthood [39]. Testosterone has been shown to reduce interferon-γ (IFN-γ) and interleukin-4 (IL-4). These are vital cytokines for immune defenses and play key roles in balancing immune responses to maintain proper functioning [39]. For example, studies using mice have shown a Th1/Th2 cell imbalance following exposure to lead [17]. In addition, given its sensitivity to perturbation during developmental period in gestation and through early-life, lead may impact the immune system in a similar way that it effects the nervous system [40]. Many neurodevelopmental outcomes have been associated with lead exposure in early life, and a handful of studies have reported greater neurodevelopment effects among male children [33,41]. One study of 965 pregnant women and their infants measured prenatal lead exposure and neurodevelopment at 6, 12, 24, and 36 months of age [41]. Greater reductions in the psychomotor development index (PDI) at 6, 12, 24, and 36 months of age among males, compared with females, associated with greater prenatal lead exposure were observed [41]. Another study of pregnant women from the ALSPAC cohort in the UK observed lower verbal, performance, and total IQ scores at 30 months of age among males, compared with females in relation to prenatal lead exposure [33]. Overall, this finding of potential sex differences is novel and future studies should consider sex when investigating lead exposure and immune function.

Although this study has some limitations it, is an important question to investigate given ubiquitous low-level lead that many children are exposed to in the United States, and lead’s potential for adversely impacting the proper functioning of the body’s main defense against infectious disease. One limitation is due to missingness of these data, which reduced the sample size. Sample size was further reduced when linking all of the various data sources. This diminished the statistical power to detect smaller differences between lead values and risk of influenza and RSV diagnosis. In addition, a selection bias may have been introduced when linking all data sources. The medical records which matched the original study sample with the SPDS birth certificate data were only for children who were born at SMH. This limits generalizability, and may potentially have induced a selection bias, as this specific group of children may be different than the total population of children tested for influenza and RSV URMC. Several sensitivity analyses were conducted to investigate these concerns, the results found the same conclusions as our primary and secondary analyses. There was also potential for exposure misclassification as lead values were rounded up or down by laboratory technicians, in an attempt to reduce this potential bias only categorical parameterizations of lead were considered. In addition, it is not believed that this misclassification would be differential by influenza or RSV diagnosis and so the results may be attenuated towards the null. The association between lead and influenza diagnosis among those who had a vaccine listed in their medical records (N = 238) was investigated. Unfortunately, the sample size became too small when considering diagnosis, various levels of lead, and other covariables. Ultimately, the current study was not able to examine vaccination status, especially while also considering covariables of interest, or divide the vaccinations up by the proper year of influenza testing (results not shown), and so investigation of vaccine effectiveness in any way was not feasible. It is not believed that vaccination would be a confounder given it should not be associated with blood lead measurements. Given vaccination is mostly a preventative tool to decrease influenza infection, by not including vaccination in the analyses, results were likely lacking some additional precision. However, the effect estimate is believe to be accurate. There is potential for residual confounding in the current study given the use of existing medical data. Additional maternal factors, such as maternal comorbidities, were not available in the child’s medical record. However, these maternal comorbidities would not likely impact childhood lead exposure directly. Thus, it is unlikely these additional maternal factors are confounders for the association of interest. Additionally, our sensitivity analyses examining the minimally adjusted models consistently reported stronger effect estimates for the association between BLLs and influenza, compared with the fully adjusted models. This suggests the additional covariables included in the fully adjusted model, including all the maternal factors and area-level characteristics, were inducing positive confounding and were important to include in the models to obtain unbiased estimates. Lastly, experimental literature has suggested greater lead toxicity during gestational exposure, in the present study only postnatal exposure was evaluated. Future work should examine the relationship between gestational lead exposure and immune function in infants and children, because this is a critical time for immune system development.

The current study had many strengths including that it was the first to examine the relationship between lead and respiratory infections. Several studies have examined hypersensitivity outcomes such as asthma and allergy. Immune suppression has only been analyzed by investigating reductions in varying cell types such as total T cells, B cells, CD4 and CD8 cells, and non-specific antibody levels. However, it is not known whether the reductions observed of these varying cell types in these studies would ultimately cause disease. Additionally, lab confirmed cases and non-cases of influenza and RSV were analyzed, because of this there was no outcome misclassification in the present study as the sensitivity and specificity of the viral test used is high. Lab confirmed blood lead levels available were utilized, and so no proxy measure for lead (i.e., housing) was needed. To test the hypotheses, a test-negative case-control study was conducted, which is considered the gold standard observational study design to assess vaccine effectiveness. The purpose of selecting only children who were tested for these respiratory infections is the underlying assumption that this population was exposed to influenza or RSV virus. Children are only tested by viral testing if the physician suspects a potential diagnosis by observing certain respiratory symptoms or automatically receives a test during the influenza season (because it is assumed exposure to influenza is high). Thus, the underlying assumption is that children who exhibit these respiratory symptoms have been exposed to influenza or RSV virus, but not all contract the disease. Although this is a crude way to attempt equal risk of exposure to influenza or RSV in this study population, it was the best way to do so in a retrospective, observational setting [42]. Lastly, the current study considered many confounders when estimating the association between lead and RSV/influenza infection. Previous studies examining lead and immune biomarkers did not consider any covariables, or only included age, and a few others. The present analysis included as many potential demographic, maternal, and socioeconomic risk factors for infection and confounders as were available.

## 5. Conclusions

In conclusion, there is no association between lead exposure and RSV and influenza disease in young children, when considering all sexes in the current study. There was, however, an observed association between blood lead levels and influenza diagnosis among males, specifically. These conclusions are made cautiously as there were several limitations in this study. Future research should be done to estimate the association between low-level lead exposure and infection risk in young children that builds on the present findings and approach while also considering sex differences. The current literature suggests increased exposure to lead, vulnerability to lead, and lead-induced immunosuppression in these young populations, as well as persistent low-level lead exposure even in lieu of lead regulations. In addition, these young children are at a large risk for infection, and infection-related complications. All of these aspects make this an important question that still needs to be investigated. Future work should also evaluate vaccine effectiveness; a prospective study to assess lead exposure, vaccination, and influenza risk is needed. This question is of tremendous public health significance and can have implications for policy change as vaccines are our only form of infectious disease prevention. If an environmental toxicant that is still abundant and common in young children influences the effectiveness of vaccines at low exposure levels, this is the reason for further regulations and enforcement of lead exposure reduction in our young populations.

## Figures and Tables

**Table 1:** Differences in demographic characteristics among children by peak lead values (N = 617) ^1^.

Characteristics	Peak Lead <3 µg/dL (N = 499) n (%)	Peak Lead ≥3 µg/dL (N = 118) n (%)	*p*-Value ^2^
***Child characteristics***			
**Age (months)**			0.8368
0.0–5.9	191 (38)	47 (40)	
6.0–11.9	119 (24)	27 (23)	
12.0–23.9	137 (27)	29 (25)	
≥24.0	52 (10)	15 (13)	
Median age (months)	9.1	8.0	
**Race**			<0.0001
White	210 (42)	32 (27)	
Black	204 (41)	80 (69)	
Other	85 (17)	6 (5)	
**Ethnicity**			0.0824
Non-Hispanic	440 (88)	97 (82)	
Hispanic	59 (12)	21 (18)	
**Insurance**			0.4282
Private	377 (76)	85 (72)	
Public/Self-Pay	122 (24)	33 (28)	
**Sex**			0.1424
Male	270 (54)	55 (47)	
Female	229 (46)	63 (53)	
***Maternal Characteristics***			
**Maternal Age (years)**			<0.0001
<20	27 (5)	17 (14)	
20–24	133 (27)	42 (36)	
25–30	143 (29)	36 (31)	
≥35	196 (39)	23 (19)	
Median age (years)	27.00	24.50	
**Parity**			0.3057
0	165 (33)	44 (37)	
1	179 (36)	32 (27)	
2	92 (18)	27 (23)	
≥3	63 (13)	15 (13)	
**Feeding Type During Hospitalization**			0.2484
Formula only	114 (23)	19 (16)	
BM and Formula	275 (55)	73 (62)	
Breastmilk only	110 (22)	26 (22)	
**Pre/Perinatal Smoker**			0.0383
No	417 (84)	89 (75)	
Yes	82 (16)	29 (25)	
***Area-Level Characteristics ^3^***			
**High Proportion Unemployment**			0.1357
No	258 (52)	52 (44)	
Yes	241 (48)	66 (56)	
**High Proportion Less than College Education**			0.0376
No	226 (45)	41 (35)	
Yes	273 (55)	77 (65)	
**High Proportion Below Poverty Level**			0.8910
No	253 (51)	59 (50)	
Yes	246 (49)	59 (50)	
**High Proportion Houses Built Before 1980**			0.3279
No	266 (53)	57 (48)	
Yes	233 (47)	61 (52)	

^1^ Samples size for children with complete data on infection, lead, and all additional covariables of interest. ^2^ Chi-square *p*-value to test for differences among all covariables and dichotomous peak lead values. ^3^ Area-level characteristics were divided by the median proportion of each variable among all census tracts in the catchment area, a high proportion is greater than the median proportion.

**Table 2:** Differences in demographic characteristics among children with and without available blood lead values (N = 1278) ^1^.

Characteristics	With Blood Lead Values	Without Blood Lead Values	*p*-Value ^2^
	(N = 617)	(N = 661)	
	n (%)	n (%)	
***Child characteristics***			
**Age (months)**			<0.0001
0.0–5.9	191 (38)	333 (50)	
6.0–11.9	119 (24)	143 (22)	
12.0–23.9	137 (27)	114 (17)	
≥24.0	52 (10)	71 (11)	
Median age (months)	9.1	6	
**Race**			<0.0001
White	210 (42)	490 (74)	
Black	204 (41)	124 (19)	
Other	85 (17)	47 (7)	
**Ethnicity**			0.0112
Non-Hispanic	440 (88)	604 (91)	
Hispanic	59 (12)	57 (9)	
**Insurance**			0.0647
Private	377 (76)	524 (79)	
Public/Self-Pay	122 (24)	137 (21)	
**Sex**			0.4102
Male	270 (54)	364 (55)	
Female	229 (46)	297 (45)	
***Maternal Characteristics***			
**Maternal Age (years)**			<0.0001
<20	27 (5)	21 (3)	
20–24	133 (27)	106 (16)	
25–30	143 (29)	161 (24)	
≥35	196 (39)	373 (56)	
Median age (years)	27	30	
**Parity**			0.6794
0	165 (33)	231 (35)	
1	179 (36)	208 (31)	
2	92 (18)	140 (21)	
≥3	63 (13)	82 (12)	
**Feeding Type During Hospitalization**			<0.0001
Formula only			
BM and Formula	114 (23)	219 (33)	
Breastmilk only	275 (55)	319 (48)	
	110 (22)	123 (19)	
**Pre/Perinatal Smoker**			0.7529
No	417 (84)	547 (83)	
Yes	82 (16)	114 (17)	
***Area-Level Characteristics*^3^**			
**High Proportion Unemployment**			0.0483
No	258 (52)	295 (45)	
Yes	241 (48)	366 (55)	
**High Proportion Less than College Education**			0.0003
No	226 (45)	354 (54)	
Yes	273 (55)	307 (46)	
**High Proportion Below Poverty Level**			0.1492
No	253 (51)	306 (46)	
Yes	246 (49)	355 (54)	
**High Proportion Houses Built Before 1980**			0.94
No	266 (53)	347 (53)	
Yes	233 (47)	314 (47)	

^1^ Sample size for all children with complete data on infection, and all covariables of interest. Among this sample, differences were examined by those with and without available blood lead levels in medical records. ^2^ Chi-square *p*-value to test for differences among all covariables and dichotomous peak lead values. ^3^ Area-level characteristics were divided by the median proportion of each variable among all census tracts in the catchment area, a high proportion is greater than the median proportion.

**Table 3:** Crude and adjusted risk estimates of respiratory syncytial virus (RSV) comparing cases with controls by peak blood lead concentrations among larger and reduced samples.

Peak Lead	Larger Sample		Reduced Sample	
	^1^ n = 558/2105		^2^ n = 123/494	
	Crude OR	Adjusted OR ^3^	Crude OR	Adjusted OR ^4^
	(95% CI)	(95% CI)	(95% CI)	(95% CI)
**LOQ Split (1 µg/dL)**				
<LOQ	1	1	1	1
≥LOQ	1.17 (0.97, 1.42)	1.19 (0.98, 1.46)	0.97 (0.65, 1.45)	0.95 (0.60, 1.49)
**3 µg/dL cut-point**				
<3 µg/dL	1	1	1	1
≥3 µg/dL	1.16 (0.92, 1.46)	1.15 (0.90, 1.46)	0.97 (0.58, 1.60)	0.93 (0.53, 1.63)
**Categories**				
<1 µg/dL	1	1	1	1
≥1 to <3 µg/dL	1.15 (0.92, 1.44)	1.18 (0.93, 1.49)	0.97 (0.59, 1.60)	0.97 (0.56, 1.66)
≥3 µg/dL	1.21 (0.95, 1.53)	1.22 (0.94, 1.57)	0.96 (0.57, 1.62)	0.90 (0.50, 1.62)

^1^ Sample size for all cases/controls included in analyses with the larger, minimally adjusted sample. ^2^ Sample size for all complete cases/controls included in analyses with reduced, fully adjusted sample. ^3^ Adjusted for only covariables available in medical records. These include child age ^2^, child sex, child race, child ethnicity, insurance status, and respiratory season. ^4^ Fully adjusted for child age ^2^, child sex, child race, child ethnicity, insurance status, maternal age, parity, feeding type, maternal smoking before and/or during pregnancy, respiratory season, area-level below poverty status, area-level unemployment status, area-level less than college education, area-level housing built before 1980.

**Table 4:** Crude and adjusted risk estimates of influenza comparing cases with controls by peak blood lead concentrations.

Peak Lead	Larger Sample		Reduced Sample	
	^1^ n = 209/2454		^2^ n = 49/568	
	Crude OR	Adjusted OR ^3^	Crude OR	Adjusted OR ^4^
	(95% CI)	(95% CI)	(95% CI)	(95% CI)
**LOQ Split (1 µg/dL)**				
<LOQ	1	1	1	1
≥LOQ	1.54 (1.16, 2.05)	1.53 (1.13, 2.08)	1.59 (0.89, 2.85)	1.34 (0.65, 2.75)
**3 µg/dL cut-point**				
<3.00 µg/dL	1	1	1	1
≥3.00 µg/dL	1.36 (0.97, 1.89)	1.33 (0.93, 1.89)	1.25 (0.62, 2.52)	0.92 (0.40, 2.14)
**Categories**				
<1 µg/dL	1	1	1	1
≥1 to <3 µg/dL	1.53 (1.10, 2.15)	1.51 (1.06, 2.16)	1.70 (0.85, 3.39)	1.52 (0.69, 3.37)
≥3 µg/dL	1.57 (1.09, 2.22)	1.56 (1.07, 2.29)	1.47 (0.70, 3.10)	1.12 (0.45, 2.82)

^1^ Sample size for all cases/controls included in analyses with the larger, minimally adjusted sample. ^2^ Sample size for all complete cases/controls included in analyses with the reduced, fully adjusted sample. ^3^ Adjusted only for covariables available in medical records. These include child age ^2^, child sex, child race, child ethnicity, insurance status, and respiratory season. ^4^ Fully adjusted for child age ^2^, child sex, child race, child ethnicity, insurance status, maternal age, parity, feeding type, maternal smoking before and/or during pregnancy, respiratory season, area-level below poverty status, area-level unemployment status, area-level less than a college education, area-level housing built before 1980.

**Table 5:** Adjusted risk estimates of respiratory syncytial virus (RSV) and influenza comparing cases with controls by peak blood lead concentrations stratified by sex (N = 2663).

	RSV Adjusted OR^1^ (95% CI)		Influenza Adjusted OR ^1^ (95% CI)	
Peak Lead	Males	Females	Males	Females
	^2^ n = 303/1141	^2^ n = 255/964	^2^ n = 116/1328	^2^ n = 93/1126
**LOQ Split (1 µg/dL)**				
<LOQ	1	1	1	1
≥LOQ	1.09 (0.82, 1.43)	1.34 (0.99, 1.81)	1.89 (1.25, 2.86)	1.17 (0.74, 1.86)
**3 µg/dL cut-point**				
<3.00 µg/dL	1	1	1	1
≥3.00 µg/dL	1.31 (0.95, 1.81)	0.99 (0.68, 1.44)	1.43 (0.90, 2.28)	1.13 (0.65, 1.97)
**Categories**				
<1 µg/dL	1	1	1	1
≥1 to <3 µg/dL	0.93 (0.66, 1.31)	1.48 (1.05, 2.07)	1.93 (1.19, 3.12)	1.15 (0.68, 1.96)
≥3 µg/dL	1.28 (0.91, 1.80)	1.15 (0.77, 1.72)	1.84 (1.11, 3.06)	1.19 (0.66, 2.17)

^1^ Adjusted for child age ^2^, child race, child ethnicity, child insurance, and respiratory season. ^2^ Sample sizes for cases/controls with RSV or influenza among males or females.

**Table 6:** Crude and adjusted risk estimates of influenza comparing cases and controls by peak blood lead concentrations in the reduced study sample, while comparing adjustment of ONLY child characteristics and full adjustment of all covariables (N = 617).

Peak Lead	Influenza	No Influenza	Crude RR	Adjusted RR ^2^ (95% CI)	Adjusted RR ^3^ (95% CI)
	N = 49	N = 568	(95% CI)	N = 49/568	N = 49/568
	n (%)	n (%)	^1^ N = 49/568		
**LOQ Split (1 µg/dL)**					
<LOQ	24 (49)	343 (60)	1	1	1
≥LOQ	25 (51)	225 (40)	1.59 (0.89, 2.85)	1.93 (1.02, 3.67)	1.34 (0.65, 2.75)
**3 µg/dL cut-point**					
<3.00 µg/dL	38 (78)	461 (81)	1	1	1
≥3.00 µg/dL	11(22)	107 (19)	1.25 (0.62, 2.52)	1.42 (0.66, 2.04)	0.92 (0.40, 2.14)
**Categories**					
<1 µg/dL	24 (49)	343 (60)	1	1	1
≥1 to <3 µg/dL	14 (29)	118 (21)	1.70 (0.85, 3.39)	2.00 (0.96, 4.16)	1.52 (0.69, 3.37)
≥3 µg/dL	11 (22)	107 (19)	1.47 (0.70, 3.10)	1.84 (0.81, 1.11)	1.12 (0.45, 2.82)

^1^ Sample size for all complete cases/controls included in analyses. ^2^ Minimally adjusted for child age ^2^, child race, child ethnicity, child insurance, and respiratory season. ^3^ Fully adjusted for age ^2^, race, ethnicity, insurance, parity, feeding, maternal smoking before and during pregnancy, respiratory season, area-level below poverty status, area-level unemployment status, area-level less than college education, area-level housing built before 1980.

## Supplementary Materials

The following are available online at https://www.mdpi.com/1660-4601/17/20/7625/s1, Table S1: Differences in demographic characteristics among full sample of children with viral testing and reduced sample of children with complete data from all sources (medical records, SPDS, and US census), also considering the availability of blood lead levels (BLLs).
