# Supplementary material for: Environmental Lead Exposure and Influenza and Respiratory Syncytial Virus Diagnoses in Young Children: A Test-Negative Case-Control Study
Source: Int J Environ Res Public Health. 2020 Oct 19;17(20):7625. doi: 10.3390/ijerph17207625 (PMC7590174; doi:10.3390/ijerph17207625)
Supplement: Supplementary file 1 [file ijerph-17-07625-s001.pdf]

Supplementary Materials:

Table S1. Differences in demographic characteristics among full sample of children with viral testing and reduced sample of children with complete data from all sources (medical records, SPDS, and US census), also considering availability of blood lead levels (BLLs)

Characteristics	All children with viral tests and available BLLs (N=2,663) n (%)	Reduced sample of children with available BLLs (N=617) n (%)	All children with viral tests and no available BLLs (N=4,466) n (%)	Reduced sample of children without available BLLs (N=661) n (%)
Child characteristics				
**Age (months)**				
0.0-5.9	826 (31)	191 (38)	1790 (40)	333 (50)
6.0-11.9	573 (22)	119 (24)	803 (18)	143 (22)
12.0-23.9	742 (28)	137 (27)	1010 (23)	114 (17)
≥24.0	522 (20)	52 (10)	863 (19)	71 (11)
Median age (months)	11.2	9.1	8.8	6.0
**Race**				
White	1142 (43)	210 (42)	3454 (77)	490 (74)
Black	1143 (43)	204 (41)	618 (14)	124 (19)
Other	378 (14)	85 (17)	394 (9)	47 (7)
**Ethnicity**				
Non-Hispanic	2322 (87)	440 (88)	4185 (94)	604 (91)
Hispanic	341 (13)	59 (12)	281 (6)	57 (9)
**Insurance**				
Private	1921 (72)	377 (76)	3252 (73)	524 (79)
Public/Self-Pay	742 (28)	122 (24)	1213 (27)	137 (21)
**Sex**				
Male	1444 (54)	270 (54)	2544 (57)	364 (55)
Female	1219 (46)	229 (46)	1922 (43)	297 (45)
